# Complement sC5b-9 and CH50 increase the risk of cancer-related mortality in patients with non-small cell lung cancer

**DOI:** 10.7150/jca.46721

**Published:** 2020-10-18

**Authors:** Jing Li, Zhijun Cao, Lijie Mi, Zhihua Xu, Xiangmei Wu

**Affiliations:** 1 Department of Endocrinology, Suzhou Xiangcheng People's Hospital, Suzhou, China; 2 Department of Medicine, Respiratory, Emergency and Intensive Care Medicine, The Affiliated Dushu Lake Hospital of Soochow University, Suzhou, China; 3 Department of General Surgery, The First Affiliated Hospital of Soochow University, Suzhou, China; 4 Department of Urology, The Ninth People's Hospital of Suzhou, Suzhou, China; 5 Department of Cardiovascular, The First Affiliated Hospital of Soochow University, Suzhou, China

**Keywords:** non-small cell lung cancer, sC5b-9, CH50, overall survival, prediction model

## Abstract

**Objectives:** Immunologic dysfunction occurred in most of patients with non-small cell lung cancer (NSCLC), which worsened the overall survival (OS) of patients. Complement activation plays a significant role in abnormal activation of immune system. However, the prognostic value of complement components such as CH50 and sC5b-9 in NSCLC patients remains unclear. This study evaluated the risk factors of NSCLC and created a prediction model.

**Methods:** A real-world study was conducted including data from 928 patients with NSCLC between April 1, 2005 and June 1, 2015. CH50 and sC5b-9 were recorded during the admission. Cox proportional hazard model was applied for survival analyses and for assessing risk factors of cancer-related mortality and to create a nomogram for prediction. The accuracy of the model was evaluated by C-index and calibration curve.

**Results:** In this study, the mortality in group with high CH50 level (≥ 480.56 umol/L) was 92.0%. Based on univariate analysis, we put factors (*P* <0.05) into a multivariate regression model, patients with high CH50 level (*P* <0.001, HR=1.59) and sC5b-9 >1422.18 μmol/L (*P* <0.001, HR=2.28) remained statistically factors for worsened OS and regarded as independent risk factors. These independently associated risk factors were applied to establish an OS estimation nomogram. Nomogram revealed good accuracy in estimating the risk, with a bootstrap-corrected C index of 0.741.

**Conclusion:** sC5b-9 and CH50 increased the risk of cancer-related mortality in patients with NSCLC. Nomogram based on multivariate analysis demonstrated good accuracy in estimating the risk of overall mortality.

## Introduction

Lung cancer is a fatal malignant tumor, and its incidence rate and mortality rate have been greatly improved internationally 1. Previous reports have demonstrated that about 1.82 million cases were identified as lung cancer, with 1.59 million deaths in 2012 2. Non-small cell lung cancer (NSCLC) is the most common lung cancer, accounting for 85% of all lung cancer types 3. NSCLC mainly includes adenocarcinoma (AC) and squamous cell carcinoma (SCC) 4. At present, the treatment of NSCLC mainly includes surgery, chemotherapy and radiotherapy 5. However, due to the late diagnosis and high rate of recurrence and metastasis, the 5-year survival rate is only 16% 6. Therefore, timely diagnosis is important to improve the survival rate of lung cancer patients.

Complement is a key factor in the innate immune immunity and in the maintenance of host homeostasis 7. It consists of more than 50 plasma proteins produced mainly by the liver as well as receptors expressed on the membranes of different kinds of cells 8. The complement system can be activated via classical, lectin, or alternative pathways, all of which unite at the level of central complement component C3 activation, leading to the formation of the membrane attack complex (MAC) and to cell lysis 9. Recent discoveries have made it clear that complement proteins exist in the tumor microenvironment and that malignant cells have the ability to produce in situ a large spectrum of the components 10, 11.

A solid body of evidence has accumulated to reveal that the complement system plays a role in cancer cell growth by promoting proliferation, angiogenesis, and antitumor immunity 12. Evidence supporting complement activation associated with C5b-9 deposition during the antitumoral response exists in various human malignancies 13. Niculescu et al. provided the first support for the presence of C5b-9 deposition in breast cancer 14. However, the correlation of serum complement level and prognosis of patients with NSCLC remains unclear.

In this study, we aimed to detect the variation in expression levels of serum complement sC5b-9 and CH50 in patients with NSCLC and explored the potential prognostic factors for NSCLC. We also showed a nomogram that could provide individualized, evidence-based, highly accurate risk estimates. Nomograms were easy to performed and could facilitate management-related decision making.

## Materials and methods

### Study design and patient characteristics

**Figure 1:**
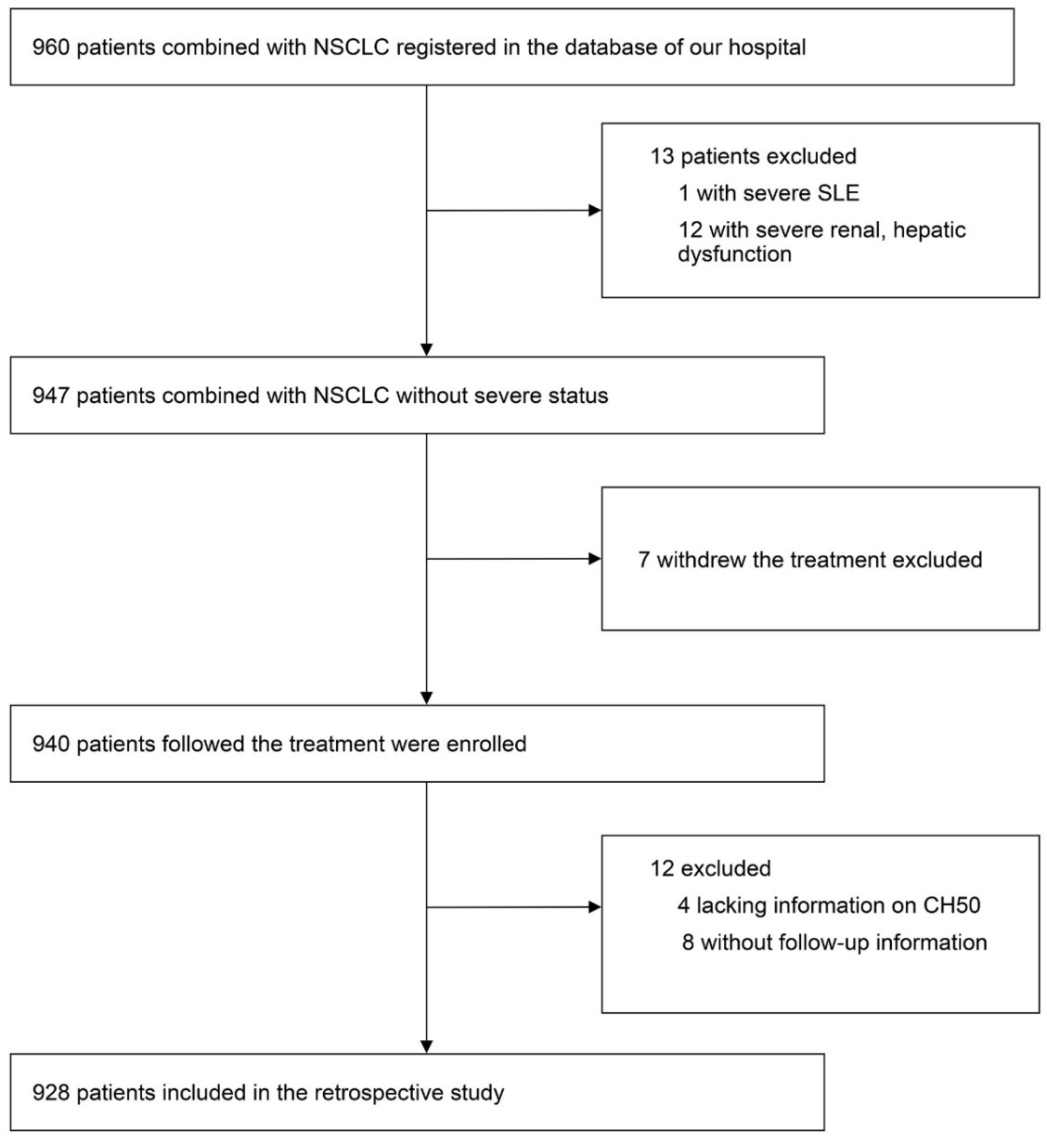
A flow chart of the screening process.

We did a real-world and retrospective study, including data from 928 patients with NSCLC between April 2005 and June 2015, in the First Affiliated Hospital of Soochow University. Those who withdrew from treatment, lacked information on complement components data or had no follow-up information were excluded. A flow chart of the screening process was shown in Figure 1. Patients' gender, age, BMI, serum CEA level, albumin level, C-reactive protein (CRP) level, lymphocytes count, neutrophils count, hemoglobin level, platelet count, PNI score, neutrophil lymphocyte ratio (NLR), stage of NSCLC, pathologic type, surgery, therapy of radiation, target therapy, application of platinum, application of vascular endothelial growth factor (VEGF) inhibitor, application of Tyrosine Kinase Inhibitor (TKI), Karnofsky Performance Status (KPS) score, smoking, heart failure, diabetes, acute coronary syndrome (ACS), and hyperlipemia were recorded. Diagnosis of NSCLC was confirmed by histopathological examination. The definition and details of all the variables above were provided in Supplemental Materials Part I. The median length of follow-up was 23.6 months. Inform, and consent was obtained from all patients or their immediate family members. All protocols were in line with the guidelines of the ethic committee of the First Affiliated Hospital of Soochow University, and following the Declaration of Helsinki.

### Assays for plasma sC5b-9 and CH50 levels

Peripheral blood samples of NSCLC patients were collected when diagnosed. Then, all of the samples were treated with EDTA as an anticoagulant, and centrifuged at 800 × g for 10 min at room temperature to collect the plasma. Next, all the plasma samples were stored at -80℃ for further research. Plasma levels of sC5b-9 and CH50 were detected by ELISA kits (Xitang, Shanghai, China) according to the manufacturer's instruction.

### Statistical analysis

Sample size assessment was performed using NCSS-PASS software version 11.0 (https://www.ncss.com/software/pass/). Power was set as 0.99, and alpha was 0.5. The mortalities of both CH50 high-level group and CH50 low-level group in our previous data (2008-2009) (0.600 and 0.950) were entered into the PASS. The Actual Hazard Ratio was set as 1.50. Then the sample size was calculated via PASS, and the minimum sample size was 330 (control = 165, experiment = 165). Our sample size was 928 (464 for each group), which was suitable. The report of the sample size assessment was displayed in Supplemental Material Part II. The missing data (<5.0%) were estimated by random forest algorithm using the mice package in RStudio (R version 3.6.1). Categorical variates were presented as percentages and compared via the κ² test. Continuous variates with skewed and normal distributions were presented as median with interquartile ranges and mean ± standard deviation. The Mann-Whitney U test and the unpaired t-test were used for comparison between Groups. Cumulative mortality was displayed by the Kaplan-Meier curve and analyzed by the log-rank test. Univariate and multivariate survival analyses for OS were assessed using the Cox regression model. The forest plots were applied to visualize the significance of covariates to the prognosis. The restricted cubic spline analyses were conducted with Harrell's Regression Modelling Strategies (rms) package.

To establish a prognostic risk model, the least absolute shrinkage and selection operator (LASSO) method, which is suitable for the regression of high-dimensional data, was performed to identify risk factors associated with prognosis. The contribution of each covariate was quantified and visualized in a prognostic nomogram with internal validation through 1000-times bootstrapping. The consistency of the resulting model was evaluated by the calibration assay. Decision curve analyses were used to assess net clinical benefits of the model compared with conventional prognostic scores. The scatter plots were used for visualization of the consistency of each model. A 1000-time bootstrapping was employed as indicated. Kaplan-Meier curves and log-rank test were applied to analyze the correlation of CH50, sC5b-9 class with survival endpoints. Statistical analysis was conducted using the RStudio (R version 3.6.1) with the following packages: 'ggplot2', 'rms', 'PredictABLE', 'risk regression', and 'survminer'.

## Results

### Baseline characteristics

During the study period, a total of 928 NSCLC patients who were diagnosed between April 2005 and June 2015 were included. The median age was 64 years old (58-69), and it contains 630 (68.0%) males. Median serum CEA and CRP level was 3.35 ng/ml and 6.46 μmol/L respectively. The pathologic type of these patients was as follow: 631 (68.0%) with adenocarcinoma, 6 (1.0%) with mixed lung cancer, 42 (5.0%) with large cell lung cancer, 242 (26.0%) with squamous carcinoma, and 7 (1.0%) with other types. For the stage of NSCLC, 136 (15.0%) patients were diagnosed with stage I, 78 (8.0%) patients were stage II, 207 (22.0%) were stage III, while 507 (55.0%) patients got stage IV. 356 (38.0%) patients accepted the surgery therapy, and 307 (33.0%) patients got the therapy of radiation. Platinum was applied to 895 (96.0%) patients and TKI was used for 266 (28.0%) patients. We also estimated the KPS score of these patients, and 584 (62.9%) patients got 80 or higher points. Basic diseases were also assessed in these patients. Diabetes was found in 91 (9.0%) patients, while hyperlipemia was 80 (9.0%). Cardiovascular diseases such as heart failure and ACS were found in 10 (1.0%) and 18 (2.0%) patients, respectively. 351 (38.0%) patients suffered from hypertension. In addition, 457 (49.0%) had a habit of smoking among all of the patients. The baseline characteristics of these patients were listed in Table 1.

Among all the 928 patients, the overall mortality was 78.5%. The mortality in high CH50 level group was 92.0%, while the low group was 65.0%. Moreover, in the high CH50 level group, patients with stage III or IV were 382 (82.0%), while the low group was 332 (71.0%) (Table 1).

### Plasma sC5b-9 and CH50, and clinical risk factors predict the development of NSCLC

**Figure 2:**
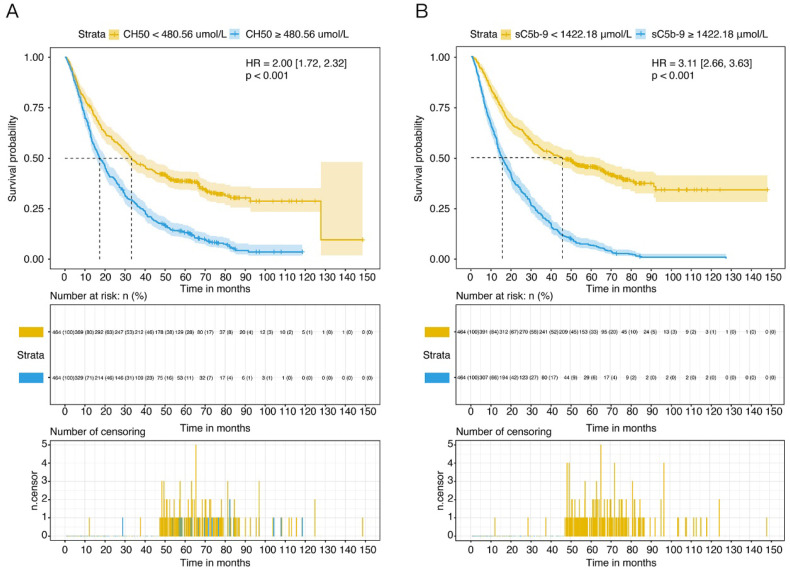
Overall survival (OS) of NSCLC patients in different levels of complement components. (A) OS of NSCLC patients with high or low level of CH50. (B) OS of NSCLC patients with high or low level of sC5b-9.

According to the univariate analysis, high CH50 levels (≥ 480.56 μmol/L) was a strong predictor of cancer-related mortality (HR 1.59, 95% CI 1.36-1.85, *P* <0.001) (Table 2). Kaplan-Meier curve displayed that patients with CH50 high levels class had increased cumulative incidence of death compared to those with the CH50 low levels class (log-rank *P* <0.001) (Figure 2A). Meanwhile, patients who had high level of sC5b-9 (≥ 1422.18 μmol/L) also showed a high morality compared to those patients with low levels group in the survival curve (HR 3.11, 95% CI 2.66-3.63, *P* <0.001) (Figure 2B).

In addition, gender, age, BMI, serum CEA and CRP level, albumin level, neutrophils and platelet counts, PNI score, NLR, metastasis, stage of NSCLC, surgery, therapy of radiation, application of platinum or TKI, target therapy, chemotherapy, smoking, hyperlipemia, heart failure, and KPS score were also associated with overall mortality (Table 2). When adjusted by age and gender, CH50 (HR 2.01, 95% CI 1.73-2.33, *P* <0.001) and sC5b-9 (HR 3.02, 95% CI 2.58-3.53, *P* <0.001) high level also displayed a high cumulative incidence of death compared to those with the low level classes.

### Independent prognostic factors for OS of NSCLC patients

**Figure 3:**
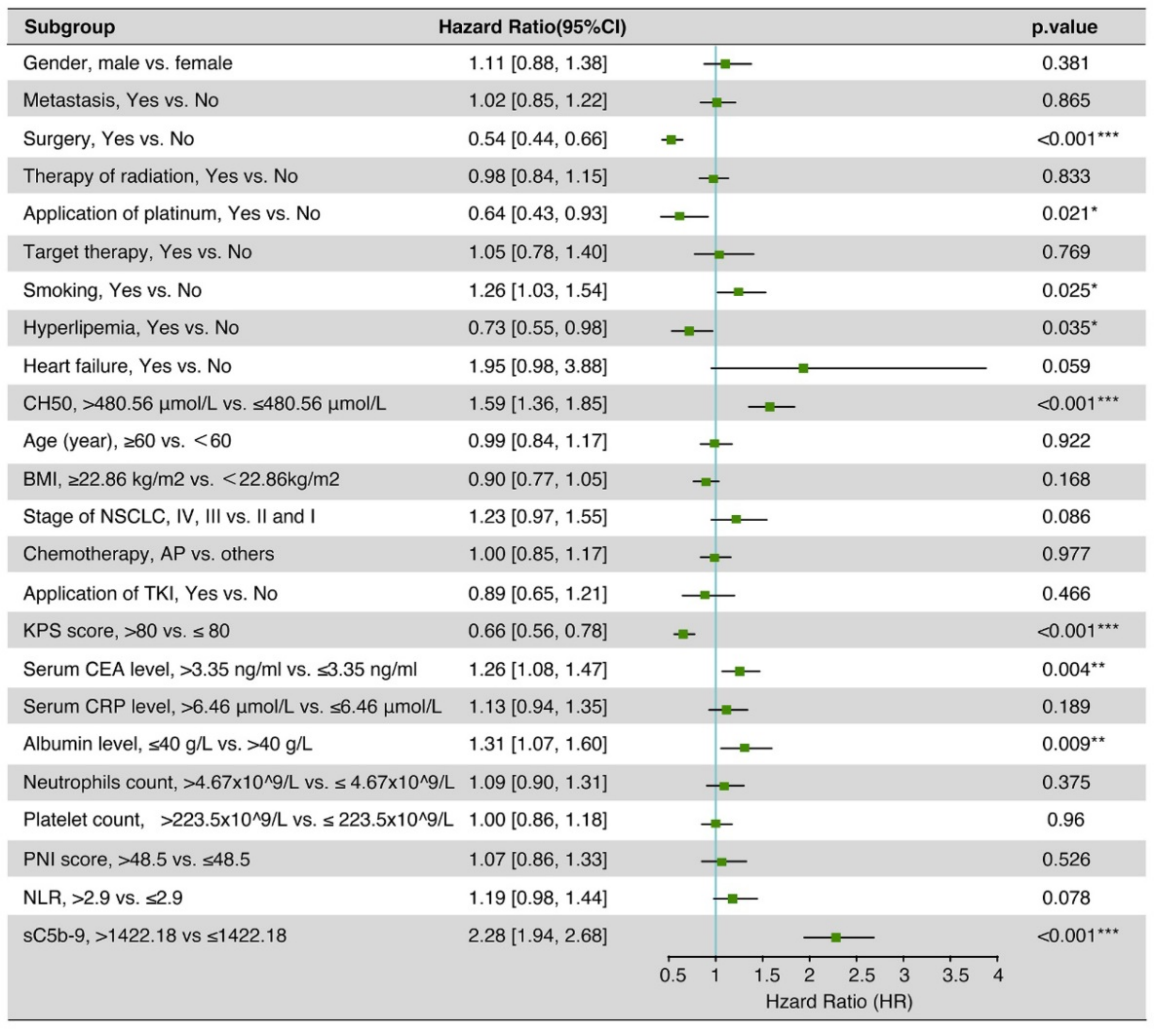
Multivariate cox regression analysis of 5-year overall survival on data in the patients with NSCLC.

After multivariate adjustment, the sC5b-9 (HR 2.28, 95% CI 1.94-2.68, *P* < 0.001) and CH50 (HR 1.59, 95% CI 1.36-1.85, *P* <0.001) were also correlated with a high increase in the risk of death (Figure 3). Meanwhile, smoking, hyperlipemia, albumin level, serum CEA level, KPS score, surgery, and application of platinum were also the independent risk factors for OS of NSCLC patients.

### Development and validation of an OS-predicting nomogram

**Figure 4:**
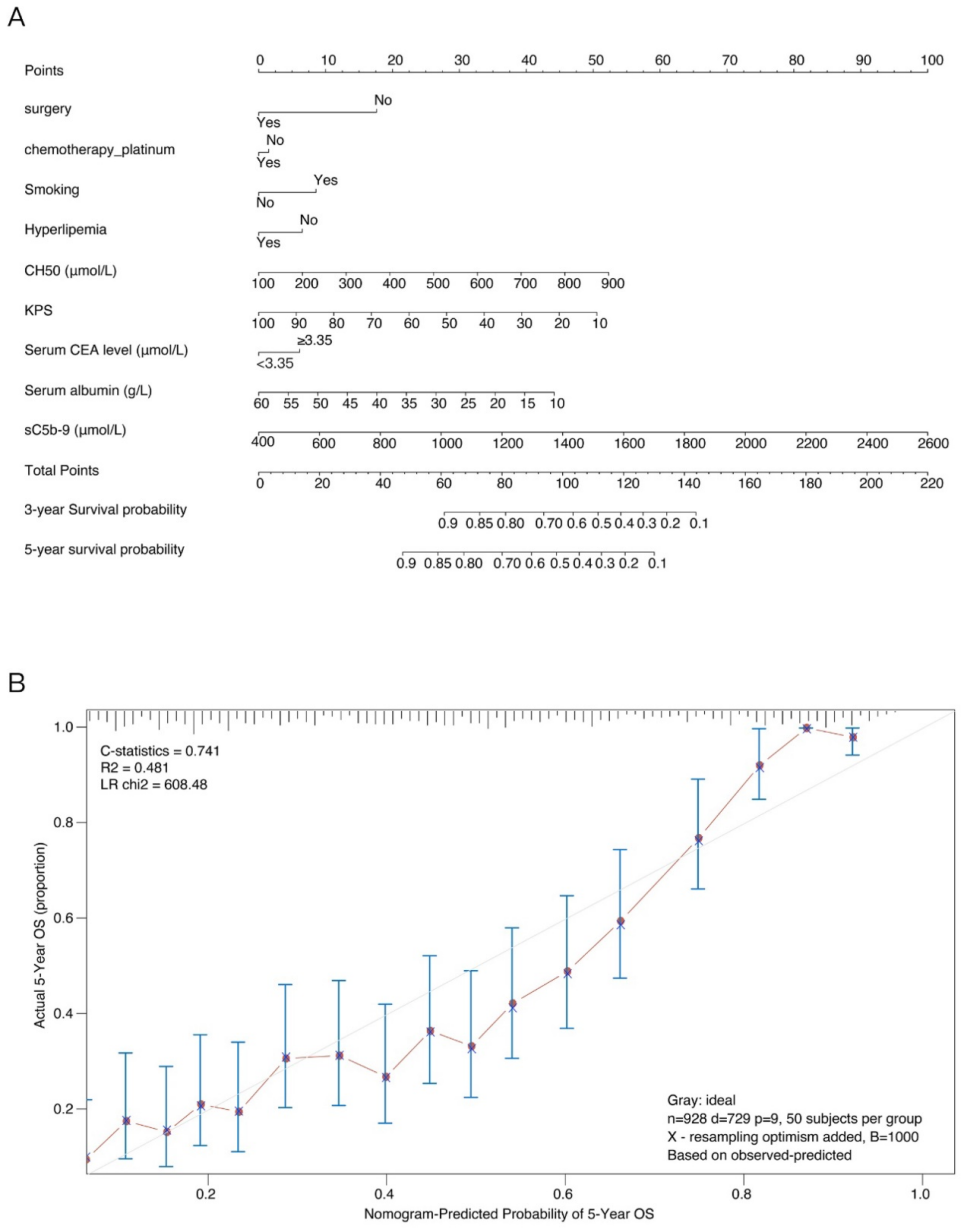
Nomogram for overall survival (OS) risk estimation of NSCLC patients and its predictive performance. (A) Nomogram to estimate the OS risk of patients with NSCLC in different variations. To build the nomogram, find the position of each variable on the corresponding axis, draw a line to the points axis for the number of points, add the points from all of the variables, and draw a line from the total points axis to determine the OS probabilities at the lower line of the nomogram. (B) Validity of the predictive performance of the nomogram in estimating the OS risk of NSCLC patients.

These independently associated risk factors that obtained from the multivariate analysis were used to form an OS estimation nomogram (Figure 4A). The prognostic model was internally validated through the bootstrap validation method. With an unadjusted C index of 0.700 and a bootstrap-corrected C index of 0.741, the nomogram displayed good accuracy in estimating the risk of OS. In the validation cohort, the nomogram showed a C index of 0.700 for the estimation of OS. A suitable calibration curve for risk estimation was also displayed (R^2^=0.481, LR chi2=608.48) (Figure 4B).

## Discussion

In this study, we detected the level of complement components, including sC5b-9 and CH50, in a large cohort of NSCLC patients at a single institution, between April 2005 and June 2015. We found that patients in high sC5b-9 or CH50 expression group had worsen OS compared with those in low sC5b-9 or CH50 expression group, respectively. The multivariate analysis indicated that sC5b-9 and CH50 were independent risk factors for OS. In addition, to propose, and retrospectively evaluate in an independent cohort, these independent prognostic factors were applied to form a nomogram for OS estimation. The nomogram suggested good accuracy in estimating the risk of OS.

Carcinogenesis involves various biological processes, including many key genes or proteins 15. The characteristics of cancer formation represent properties that a cell needs to achieve some abilities in order to become and maintain as a cancer cell 16. These hallmarks guide the immune response related to initiation and progression of cancer 17. Complement system represents a vital component of inflammatory response, which functions as a bridge linking innate and adaptive immune response 18. The contribution of complement system in the pathophysiology has been recently considered involvement in carcinogenesis 19, 20. A study investigated by Osther K et al. showed that the complement system in cancer patients may be stimulated through the complement lectin pathway 21. In addition, complement convertases C5 and C3 were found to be involved in the development of lung cancer because these 2 mediators could have an effect on classical, lectin, or alternative complement pathways 22, 23.

Previous studies have suggested that many complement components can serve as a valuable biomarker for cancer 24, 25. Recently, several complement components have been identified as a biomarker for prognostic prediction in NSCLC. Tuberk et al. found that complement components (C3 and C4) levels were abnormally expressed in lung cancer patients with multiple cell types compared to that in control group, and aberrant C3 or C4 could function as a prognostic biomarker for patients with lung cancer 26. As a member of complement components, sC5b-9 has been proved to participate in other diseases. Many studies indicated that complement system activation could give rise to some diseases through the formation of sC5b-9 27, 28. However, no studies have been conducted to explore the potential role of sC5b-9 for biomarker in cancer, especially in NSCLS. To the best of our knowledge, this study was the first attempt ever made to comprehensively elucidate the prognostic role for biomarker based on sC5b-9 or CH50 level in patients with NSCLC. In the current study, we detected the expression levels of sC5b-9 and CH50 in NSCLC patients. Moreover, the univariate analysis suggested that high CH50 level was a strong predictor for cancer-related mortality. Kaplan-Meier curve showed that patients with high sC5b-9 or CH50 level had increased cumulative incidence of death compared to those with low sC5b-9 or CH50 level, respectively. Furthermore, gender, age, BMI, serum CEA and CRP level, albumin level, neutrophils and platelet counts, PNI score, NLR, metastasis, stage of NSCLC, surgery, therapy of radiation, application of platinum or TKI, target therapy, chemotherapy, smoking, hyperlipemia, heart failure, and KPS score were correlated with overall mortality. The multivariate analysis displayed that sC5b-9, CH50, smoking, hyperlipemia, albumin level, serum CEA level, KPS score, surgery, and application of platinum were independent risk factors.

It is reported that nomograms are used for visualization of statistical models, calculation of predicted values, and graphical assessment of variable significance 29, 30. The nomograms have been widely applied for prediction of cancer risk and treatment outcomes 31, 32. Recently, some studies have successfully established prognostic nomograms that integrated miRNAs and clinical-related variables in different cancers 33-35. However, few studies have built prognostic models using combination of sC5b-9 and CH50 and clinical risk factors in NSCLC patients. In this analysis, based on the combination of sC5b-9 and CH50 and independent clinicopathological variables, we created a prognostic nomogram model that could provide an individual estimation of OS in NSCLC patients. The nomogram displayed excellent accuracy in estimating the risk of OS. The suitable calibration curve for risk estimation showed good agreements between observation and prediction. Thus, this is the first prognostic nomogram for NSCLC patients that considers clinicopathological variables in addition to sC5b-9 and CH50. Based on this model, the potential higher risk patients with low survival rate could be selected for a specific treatment.

There are some limitations existing in this study. Firstly, experiments that explore the biological implications of sC5b-9 and CH50 are needed. Therefore, the investigation of molecular mechanism of sC5b-9 and CH50 should be considered in further study in NSCLC. Secondly, the prognostic nomogram needs to be further verified by a large-scale and prospective multicenter research before it can be used in clinical practice.

Collectively, we found that sC5b-9 and CH50 were independent risk factors for prognosis prediction in patients with NSCLC. In addition, prognostic nomogram based on multivariate analysis had excellent accuracy in estimating the risk of OS.

## Supplementary Material

Supplementary details of variants and report of sample size assessment.

## Figures and Tables

**Table 1:** Study Participant Characteristics at Enrollment

Variation	Total (n=928)		Cohort, median (IQR)		
			CH50<480,(n=464)	CH50>480,(n=464)	p.value
Age, (year)	64(58,69)		63(58,69)	64(58,70)	0.19
BMI, (kg/m2)	22.86(20.44,24.86)		22.76(20.44,24.97)	22.98(20.52,24.78)	0.803
Serum CEA level, (ng/ml)	3.35(1.64,13.33)		3.18(1.49,11.73)	3.88(1.86,17.08)	0.009**
Serum CRP level, (umol/L)	6.46(1.65,12.59)		5.95(1.49,12.78)	7.28(1.88,12.47)	0.357
Albumin level, (g/L)	40(36.6,43.62)		40.1(36.6,43.52)	40(36.5,43.73)	0.915
Neutrophils count, (10^9/L)	4.67(3.46,6.27)		4.58(3.41,6.06)	4.84(3.59,6.45)	0.107
Lymphocytes count, (10^9/L)	1.61(1.25,1.97)		1.59(1.26,1.99)	1.64(1.24,1.94)	0.794
Hemoglobin level, (g/L)	132(122,142)		132(122,142.25)	132(122,142)	0.893
Platelet count, (10^9/L)	223.5(179,279)		219(174.75,275.5)	228(182,279.25)	0.131
PNI score	48.5(44.2,53.05)		48.7(44.2,52.85)	48.38(44.14,53.26)	0.781
NLR	2.9(2.07,4.31)		2.87(1.99,4.24)	2.95(2.14,4.38)	0.131
sC5b-9 (μmol/L)	1422.18(1182.69,1620.32)		1355.82(1084.96,1586.72)	1466.33(1283.25,1650.99)	<0.001***
CH50 (μmol/L)	480.56±110.07		391.11±62.73	570.01±65.46	<0.001***
C3a	570.61(488.22,650.37)		545.03(466.06,636.14)	595.89(522.21,666.22)	<0.001***
C5a	664.47±115.42		641.85±119.72	687.09±106.37	<0.001***
KPS score, IQR	90(80,90)		90(80,100)	90(80,90)	0.006**
Gender, (n%)					0.725
Female	298(32)		152(33)	146(31)	
Male	630(68)		312(67)	318(69)	
Pathologic type, (n%)					0.974
Adenocarcinoma	631(68)		314(68)	317(68)	
Mixed lung cancer	6(1)		3(1)	3(1)	
Large cell lung cancer	42(5)		23(5)	19(4)	
Squamous carcinoma	242(26)		121(26)	121(26)	
Others	7(1)		3(1)	4(1)	
Metastasis, n(%)					0.012*
No	419(45)		229(49)	190(41)	
Yes	509(55)		235(51)	274(59)	
Stage of NSCLC					0.002**
Stage I	136(15)		84(18)	52(11)	
Stage II	78(8)		48(10)	30(6)	
Stage III	207(22)		98(21)	109(23)	
Stage IV	507(55)		234(50)	273(59)	
Surgery, (n%)					<0.001***
No	572(62)		249(54)	323(70)	
Yes	356(38)		215(46)	141(30)	
Therapy of radiation, (n%)					0.026*
No	621(67)		327(70)	294(63)	
Yes	307(33)		137(30)	170(37)	
Application of platinum, (n%)					1
No	33(4)		16(3)	17(4)	
Yes	895(96)		448(97)	447(96)	
Chemotherapy					0.863
AP	436(47)		221(48)	215(46)	
DP	169(18)		78(17)	91(20)	
EP	50(5)		25(5)	25(5)	
GP	40(4)		21(5)	19(4)	
Others	233(25)		119(26)	114(25)	
Target therapy, (n%)					0.064
No	600(65)		314(68)	286(62)	
Yes	328(35)		150(32)	178(38)	
Application of TKI, (n%)					0.046*
No	662(71)		347(75)	315(68)	
TKI I	206(22)		92(20)	114(25)	
TKI II	3(0)		0(0)	3(1)	
TKI III	57(6)		25(5)	32(7)	
Application of VEGF inhibitor, n(%)					1
No	791(85)		395(85)	396(85)	
Yes	137(15)		69(15)	68(15)	
Smoking, n(%)					0.694
No	471(51)		239(52)	232(50)	
Yes	457(49)		225(48)	232(50)	
Hypertension, n(%)					0.042*
No	577(62)		304(66)	273(59)	
Yes	351(38)		160(34)	191(41)	
Diabetes, n(%)					0.508
No	837(90)		415(89)	422(91)	
Yes	91(10)		49(11)	42(9)	
Hyperlipemia, n(%)					0.726
No	848(91)		422(91)	426(92)	
Yes	80(9)		42(9)	38(8)	
Heart failure, n(%)					0.34
No	918(99)		461(99)	457(98)	
Yes	10(1)		3(1)	7(2)	
ACS, n(%)					0.812
No	910(98)		456(98)	454(98)	
Yes	18(2)		8(2)	10(2)	

Abbreviation: IQR, interquartile range; CRP, C-reactive protein; PNI, neutrophil lymphocyte ratio; NLR, neutrophil lymphocyte ratio; NSCLC, non-small-cell lung cancer; TKI, Tyrosine Kinase Inhibitor; VEGF, vascular endothelial growth factor; KPS, Karnofsky Performance Status; ACS, acute coronary syndrome. ***p<0.001, **p<0.01, *p<0.05.

**Table 2:** Cox Regression Analysis of Hazard Ratio on NSCLC patients

Variation		Non-adjustment	
		Hazard Ratio (95% CI)	p.value
Gender, male vs. female		1.53 [1.30, 1.79]	<0.001***
Age (year), ≥60 vs. <60		1.20 [1.03, 1.40]	0.022*
BMI, ≥22.86 kg/m2 vs. <22.86kg/m^2^		0.80 [0.69, 0.92]	0.002**
Serum CEA level, >3.35 ng/ml vs. ≤3.35 ng/ml		1.63 [1.41, 1.89]	<0.001***
Serum CRP level, >6.46 μmol/L vs. ≤6.46 μmol/L		1.75 [1.51, 2.03]	<0.001***
Albumin level, ≤40 g/L vs. >40 g/L		1.70 [1.46, 1.96]	<0.001***
Neutrophils count, >4.67x10^9/L vs. ≤ 4.67x10^9/L		1.59 [1.38, 1.84]	<0.001***
Lymphocytes count, >1.61x10^9/L vs. ≤ 1.61x10^9/L		0.89 [0.77, 1.03]	0.105
Hemoglobin level, >132 g/L vs. ≤ 132 g/L		0.94 [0.81, 1.08]	0.385
Platelet count, >223.5x10^9/L vs. ≤ 223.5x10^9/L		1.24 [1.08, 1.44]	0.003**
PNI score, >48.5 vs. ≤48.5		0.61 [0.53, 0.71]	<0.001***
NLR, >2.9 vs. ≤2.9		1.78 [1.54, 2.06]	<0.001***
Pathologic type, Adenocarcinoma vs. others		0.95 [0.81, 1.11]	0.526
Metastasis, Yes vs. No		1.56 [1.35, 1.81]	<0.001***
Stage of NSCLC, IV, III vs. II and I		2.00 [1.65, 2.43]	<0.001***
Surgery, Yes vs. No		0.30 [0.25, 0.35]	<0.001***
Therapy of radiation, Yes vs. No		1.21 [1.04, 1.41]	0.013*
Application of platinum, Yes vs. No		0.55 [0.38, 0.80]	0.002**
Target therapy, Yes vs. No		1.22 [1.05, 1.42]	0.009**
Application of TKI, Yes vs. No		1.20 [1.03, 1.40]	0.022*
Application of VEGF inhibitor, Yes vs. No		1.08 [0.88, 1.33]	0.442
Chemotherapy, AP vs. others		0.85 [0.74, 0.99]	0.035*
Smoking, Yes vs. No		1.61 [1.39, 1.87]	<0.001***
Hypertension, Yes vs. No		1.05 [0.90, 1.22]	0.524
Diabetes, Yes vs. No		1.07 [0.84, 1.36]	0.603
Hyperlipemia, Yes vs. No		0.59 [0.45, 0.79]	<0.001***
Heart failure, Yes vs. No		2.71 [1.40, 5.23]	0.003**
ACS, Yes vs. No		1.30 [0.78, 2.17]	0.311
KPS score, >80 vs. ≤ 80		0.43 [0.37, 0.50]	<0.001***
CH50, >480.56 μmol/L vs. ≤480.56 μmol/L		2.00 [1.72, 2.32]	<0.001***
sC5b-9, >1422.18 μmol/L vs ≤1422.18 μmol/L		3.11 [2.66, 3.63]	<0.001***

Abbreviation: HR, hazard risk; BMI, Body Mass Index; CRP, C-reactive protein; PNI, neutrophil lymphocyte ratio; NLR, neutrophil lymphocyte ratio; NSCLC, non-small-cell lung cancer; TKI, Tyrosine Kinase Inhibitor; VEGF, vascular endothelial growth factor; KPS, Karnofsky Performance Status; ACS, acute coronary syndrome. ***p<0.001, **p<0.01, *p<0.05.

## Abbreviations

IQR: interquartile range
CRP: C-reactive protein
PNI: neutrophil lymphocyte ratio
NLR: neutrophil lymphocyte ratio
NSCLC: non-small-cell lung cancer
TKI: Tyrosine Kinase Inhibitor
VEGF: vascular endothelial growth factor
KPS: Karnofsky Performance Status
ACS: acute coronary syndrome
HR: hazard risk
BMI: Body Mass Index
